# Clinical experience with ^18^F-JK-PSMA-7 when using a digital PET/CT

**DOI:** 10.1186/s41824-022-00128-3

**Published:** 2022-03-15

**Authors:** Irina Vierasu, Nicola Trotta, Simone Albisinni, Céline Mathey, Gil Leurquin-Sterk, Simon Lacroix, Gaetan Van Simaeys, Thierry Quackels, Thierry Roumeguère, Serge Goldman

**Affiliations:** 1 Nuclear Medicine Department and PET/Biomedical Cyclotron Unit, Erasme Hospital, Université Libre de Bruxelles, Route de Lennik 808, 1070 Brussels, Belgium; 2 Urology Department, Erasme Hospital, Université Libre de Bruxelles, Brussels, Belgium

**Keywords:** Prostate cancer, ^18^F-JK-PSMA-7 PET/CT, Biochemical recurrence, Detection rate

## Abstract

**Background:**

Digital PET/CT systems make use of a new technology with higher sensitivity and other better technological features than the analog ones. They require adaptation of the trade-off between performance, tracer dose and acquisition time. The aim of the study was to explore the performance of ^18^F-JK-PSMA-7 imaging when performed on a digital PET/CT with an adapted protocol, in a population of patients with prostate cancer patients (PCa). Influence of previous therapy on PET/CT performance is generally disregarded in PSMA-based imaging, despite potential influence of hormono-chemotherapy on the target expression. This potential influence was also tested in this work.

**Methods:**

A total of 54 PCa patients experiencing biochemical recurrence were included in the study, in which we analysed the diagnostic performance of digital ^18^F-JK-PSMA-7 PET/CT. Compared to our protocol applied for acquisition on an analog system, administered dose and acquisition time were reduced by 20% and 50% respectively. We specifically took into consideration the influence of previous treatments on recurrence detection.

**Results:**

We detected overall ^18^F-JK-PSMA-7-positive lesions in 38/54 patients (70.3%). There was no statistically significant difference regarding the detection rate between the groups of patients who had hormono-chemotherapy any time after initial diagnosis and those who never got any hormonal or chemotherapeutic treatment. Regarding the SUV max values, there was not significant difference between the two groups of patients neither in pelvic ganglions nor in other metastatic sites or the prostate region.

**Conclusion:**

^18F^-JK-PSMA7 PET/CT with administered dose and acquisition time adapted to the digital technology provides valuable information in PCa patients with biochemical recurrence.

## Introduction

PSMA is a class II transmembrane glycoprotein that proved to be a valuable target for radiolabeled imaging as it is significantly overexpressed in malignant prostate cells (Ghosh and Heston 2004).

PSMA imaging has been an extremely productive field over these last years, leading to a variety of positron emission tomography (PET) tracers made available for the detection of prostate cancer recurrence in clinical practice. These tracers have revolutionized the approach of prostate cancer follow up based on biochemical recurrence (BCR). Still, since the detection of malignant cells by this method relies on the overexpression of the target, the influence of anti-androgen drugs and chemotherapeutic agents on its performance remains a subject of investigations and debates.

Among the PSMA tracers, new ^18^F-labeled compounds have aroused a specific interest considering the superiority of fluorine for PET imaging as compared to the initially used ^68^ Ga. Indeed, ^18^F has the advantage of a longer half-life (i.e.109.8 min vs. 68 min), allowing for regional distribution of the tracer in a number of PET centres. Also, ^18^F exhibits lower positron energy than ^68^ Ga (0.6 MeV vs. 2.3 MeV). Therefore, the distance travelled by the positron in human tissue is much shorter, resulting in a better spatial resolution in PET images.

The efforts to take advantage of the ^18^F isotopic characteristics for PSMA imaging led to the development of ^18^F-DCFPyL as the first ^18^F-based PSMA tracer.

Several clinical trials showed that this tracer offers an excellent alternative to ^68^ Ga-PSMA for PSMA-PET/CT imaging in patients with BCR (Wondergem et al. 2019). Importantly, with high values of tumour/background ratio in PSMA-avid lesions, ^18^F-DCFPyL PET/CT provides a higher sensitivity than ^68^ Ga-based tracers for the localization of sites of recurrence, a definite asset for improved management of patients with oligometastatic recurrent prostate cancer (Dietlein et al. 2015; Ceci et al. 2015).

Among other ^18^F-labeled derivatives targeting PSMA, JK-PSMA-7, developed by the Jülich/Köln group (Forschungszentrum Jülich and the University Hospital of Köln) has a structure that only differs from that of DCFPyL by the presence of a methoxy group on the fluoropyridine ring. JK-PSMA-7 has recently been successfully tested in two clinical trials in patients with biochemical recurrent prostate cancer (Hohberg et al. 2019; Dietlein et al. 2020, 2021). Those studies were performed on a standard analog PET/CT (Dietlein et al. 2020, 2021).

The purpose of this study was to evaluate the detection efficacy of ^18^F-JK-PSMA-7 in patients with BCR when performed on a digital PET/CT with an adapted imaging protocol. Digital PET/CT systems have better technological features than those of analog systems. The improvements include a higher sensitivity, an increased signal-to-noise ratio due to a better time-of-flight (TOF) timing resolution, and better contrast recovery (Surti et al. 2007; Zhang et al. 2018). Digital PET/CT instruments also come with regularized versions of reconstruction algorithms that improve field homogeneity, by taking into account the point spread function distribution. This latter advantage impacts partial-volume effects that may lower the detectability of peripheral small lesions such as malignant pelvic lymph nodes and improves quantification accuracy. We evaluated the detection rate of our imaging procedure, with a specific attention paid to the regional lymph nodes and to the influence of clinical factors and previous treatments.

## Materials and methods

### Patients and PET data analyses

Between January 2020 and September 2020, we performed 109 PET/CT examinations using ^18^F-JK-PSMA-7 in patients referred for increased PSA blood level. We analysed clinical and imaging aspects in all 54 patients with BCR, which were followed in the Urology Department of Erasme hospital with no missing data. The retrospective analysis of data acquired on the PET/CT Vereos has been approved by the Ethics Committee of the institution, which waived the obligation of written informed consent.

The examinations were analysed by three experienced nuclear medicine physicians in a randomized order. Examinations were positive if there was at least one lesion which was considered as overexpressing PSMA based on the visual analysis of the images, in accordance to published guidelines (Ceci et al. 2021). SUVmax were calculated in all lesions detected. To calculate TBR (tumour/background ratio), the SUVmean in the left gluteal muscle was used as background value. Effect of PSA level on lesion detection was tested using a PSA threshold of 0.3 ng/ml as in a previous study performed with ^18^F-JK-PSMA-7 (Dietlein et al. 2021).

Quantitative analysis was performed using Philips software IntelliSpace Portal (ISP, Koninklijke Philips N.V., The Netherlands, version 11.1).

All statistical analyses on PET data were realized with JASP software (University of Amsterdam, The Netherlands, version 0.14.1). Group analyses were performed using unpaired *t*-tests and the results were considered significant at a *p* value < 0.05.

### ^18^F-JK-PSMA-7 tracer preparation

^18^F-JK-PSMA-7 was produced on an All-in-one automated synthesizer (Trasis SA, Belgium) configured with an HPLC purification system, as described before (Simaeys et al. 2021). All chemical reagents were provided in commercially available reagent kits by Trasis SA. The automated radiosynthesis of ^18^F-JK-PSMA-7 consists in a two-step reaction followed by an HPLC purification and a reformulation.

Briefly, ^18^F-fluoride was produced through the ^18^O(p,n)^18^F reaction in a Cyclone 30 cyclotron from IBA, Belgium. The irradiated enriched water was collected and passed through a QMA Sep-Pak carbonate cartridge where ^18^F-fluoride was trapped and [^18^O]H_2_O collected for recycling. ^18^F-fluoride is then eluted to the reactor. After ^18^F-fluoride drying at 125 °C under a stream of nitrogen, the precursor was added to the reactor and heated for 5 min at 70 °C. The reaction mixture was cooled down and hydrolysis of the protecting groups was realized. The crude mixture was diluted in saline and purification was carried out in a semi-preparative HPLC column. The resulting fraction was collected and diluted in saline and trapped for reformulation. ^18^F-JK-PSMA-7 was eluted with ethanol and formulated in a solution of sodium ascorbate in saline. Quality control of the final product was performed on each batch and complied with in-house specifications and with the principles of the general monograph on radiopharmaceutical preparations (EP 07/2016:0125). Quality controls included the assessment of appearance, pH, chemical purity, radiochemical purity and radionuclidic purity, residual solvents, bacterial endotoxins, and sterility.

### ^18^F-JK-PSMA-7 imaging

The PET/CT scans were all performed on a Philips Vereos digital PET/CT (Philips Medical Systems, Cleveland, Ohio, USA), the characteristics of which having been previously described (Zhang et al. 2018). PET acquisitions were performed 140 min post-injection based on data showing a 50% TBR increase between 100 and 140 min after tracer administration (Hohberg et al. 2019). The median injected dose of 18F-JK-PSMA-7 was 4 MBq/kg (range: 226-507 MBq). The injected dose was adapted from the dose applied for analog acquisition systems in our centre, corresponding to a reduction by 20% of the dose we have adopted for patients investigated on the Philips Gemini TF64 PET/CT (Philips Medical Systems, Cleveland, Ohio, USA). Our experience with PSMA imaging with an analog PET/CT system started in December 2017. A total of 674 examinations were performed in our centre between December 2017 and December 2019.

After the injection of the radiotracer all patients received intravenous furosemide in order to force diuresis.

A whole-body scan was performed in all patients, from skull vertex to feet. PET acquisitions were performed for approximately 14 min including a total of 17 bed positions (1 min per bed position for the 10 positions from skull to thighs and 30 s per bed position for the 7 positions from thighs to feet, adapted to the patient’s morphology). This mode of acquisition corresponds to a 50% reduction of imaging time in comparison to our protocol of acquisition on the Philips Gemini TF-64 PET/CT. PET acquisitions were combined with a low-dose non-contrast-enhanced CT (50 mAs, 120 kV). All images were corrected for radioactivity decay as well as for scatter and random coincidences; 511 keV photon attenuation correction was performed using CT images.

Images were reconstructed with the 3D ordered subset expectation maximization (OSEM) algorithm implemented on the Philips Vereos system and set up to 3 iterations, 15 subsets and a Gaussian 3D filter with a full width at half-maximum of 6 mm.

## Results

In our study population, 38 out of 54 (70.3%) patients with BCR had been treated initially by radical prostatectomy (PRT) and 4 patients by brachytherapy. A total of 32 patients in our study were pre-treated with Androgen Deprivation Therapy (ADT) among which 8 also received chemotherapy. A total of 20 patients already had a local recurrence shown on MRI and treated by radiotherapy. A total of 30 patients had a PSA level < 2 and 24 patients had a PSA level ≥ 2. The minimal PSA value was 0.12 and the median value was 1.66. Patients’ biological and clinical characteristics are presented in the Tables 1 and 2.

**Table 1:** Clinical, tumor and treatment characteristics

	PSA < 2	PSA ≥ 2	Total
Number of patients	30	24	54
Age (mean ± SD; range)	71.29 ± 6.55; 55–88	73.20 ± 8.33; 50–90	72 ± 7.43; 55–90
PSA (mean ± SD)	0.76 ± 0.56	10.26 ± 9.89	4.99 ± 8.08
PRT	22	16	38
Brachytherapy	0	4	4
Radiotherapy on the prostatic region	12	8	20
Hormonal therapy	0	32	32
Chemotherapy	0	8	8
Other treatment	0	6	6
PSMA +	16	22	38
PSMA-	14	2	16
Number of patients with lesions in prostatic region	8	7	15
Number of patients with lesions in pelvic lymph nodes	9	8	17

**Table 2:** Gleason scores and TNM staging

Gleason Score	Nb of patients
6 (3 + 3) 7 (4 + 3) 7 (3 + 4) 8 (4 + 4) 8 (3 + 5) 9 (4 + 5) 10 (5 + 5)	4 10 14 9 1 14 2

TNM	Nb of patients
T1 T2 (b/c) T3 (a,b)	1 10 43
N0 N1 N2 Nx	41 11 1 1
M0 M1	48 6

Table 3 shows the detection rate (DR) of ^18^F-JK-PSMA-7 PET/CT in our patient population and Table 4 shows the quantitative analysis in PSMA avid lesions. Overall, we detected ^18^F-JK-PSMA-7-positive lesions in 38/54 patients (70.3%). Only 2 patients of these 38 patients had a PSA level lower than 0.3.

**Table 3:** Number of lesions identified on ^18^F-JK-PSMA-7 PET/CT and detection rate

PSMA-7 PET/CT	Negative	Positive	Detection rate (DR)
PSA < 0.3	3	2	0.4
PSA ≥ 0.3	13	36	0.73
Total	16	38	0.70

**Table 4:** SUVmax in ^18^F-JK-PSMA-7 avid lesions in patients treated without and with hormono-chemotherapy, regrouped according to locations, and results of *t*-tests analyses at group level

	Prostate region	Pelvic nodes	Other lesions
Treatment without hormono-chemotherapy	nb of lesions = 4 mean = 10.4 SD = 3.68	nb of lesions = 8 mean = 23.71 SD = 25.3	nb of lesions = 4 mean = 27.82 SD = 37.5
Treatment with hormono-chemotherapy	nb of lesions = 11 mean = 10.48 SD = 5.74	nb of lesions = 16 mean = 14.26 SD = 9.91	nb of lesions = 13 mean = 20.71 SD = 22.11
Group analysis *t*-test results	*p* value = 0.979	*p* value = 0.198	*p* value = 0.639

The images showed overexpressing PSMA lesions in the prostatic area in 15 patients among which 7 having been treated by PRT, 4 having been treated by another local treatment and 4 having had no loco-regional treatment. PSMA positive lesions were found in pelvic nodes in 17 patients and in extra-pelvic nodes in 13 patients. PSMA positive bone metastases were found in 15 patients. PSMA positive lung lesions were found in 2 patients, one of which having no metastasis at time of diagnosis.

The TBR mean value for lesions in the prostatic bed was 24.91 ± 10.98 (mean ± SD), while it was 44.83 ± 46.14 in pelvic nodes.

We compared the SUVmax between the groups of patients who had hormono-chemotherapy any time after initial diagnosis and those who never got any hormonal or chemotherapeutic treatment. The SUVmax values were grouped according to the location of the PSMA positive lesions (Table 4).

Both analyses performed for the three regions taken into account did not disclose significant differences between these groups.

**Fig. 1:**
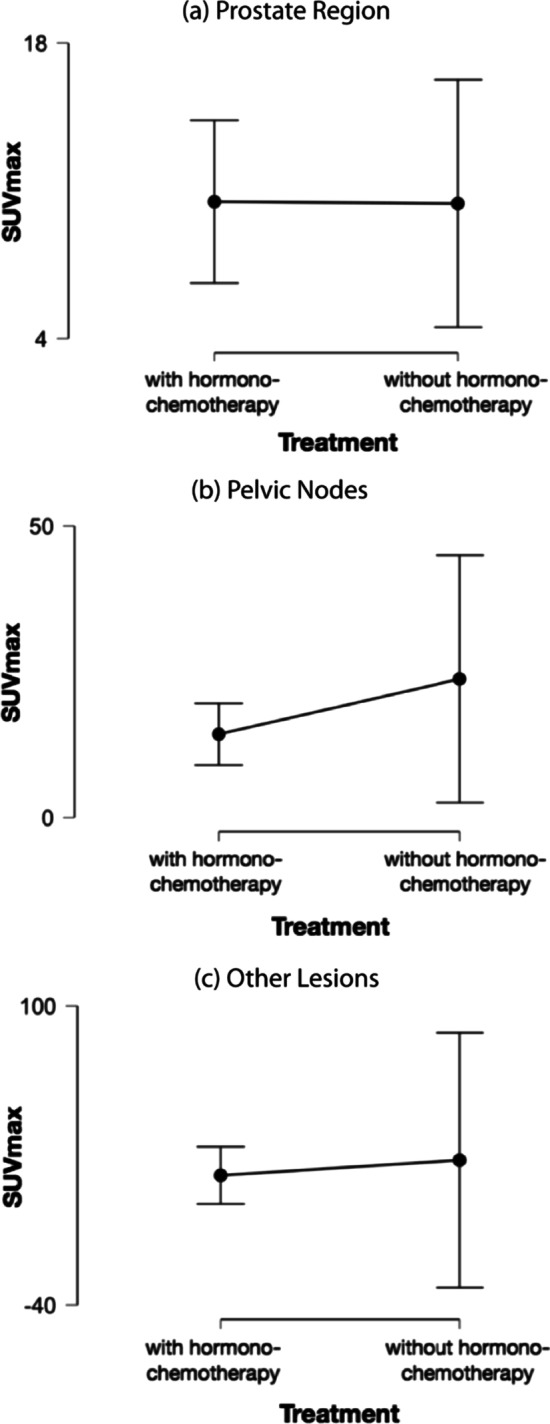
descriptive plots of *t*-tests analyses at group level

Descriptive plots of the statistical analyses are shown in Fig. 1.

## Discussion

This retrospective study showed that, when performed on a digital PET/CT with an imaging protocol adapted to the digital technology, ^18^F-JK-PSMA-7 detected lesions in 70.3% of PCa patients with suspected prostate cancer recurrence. Previous hormono-chemotherapy did not influence the performance of ^18^F-JK-PSMA-7 PET/CT in our population.

Studies on PSMA tracers in PCa patients with BCR are difficult to compare in terms of DR, considering the variable PSA levels of which it highly depends on. Indeed, in a systematic review of studies on ^68^ Ga-PSMA tracers the pooled detection rate was 70.6%. DR is not expected to be drastically higher with ^18^F-PSMA tracers, since a study comparing performances of 3 different ligands (^18^F-JK-PSMA-7, ^18^F-DCFPyL and ^68^ Ga-PSMA-11) did not demonstrate significant differences in terms of DR (Dietlein et al. 2021). DCFPyL is the tracer of this category for which the largest experience has been acquired. In the studies reporting detection rates in populations with median PSA values < 2 ng/ml—comparable to our median PSA value of 1.66-, the detection rate was 21/31 (67.7%) and 28/34 (77.8%) respectively (Ceci et al. 2021; Wondergem et al. 2017). A higher DR of 90% has been reported with the tracer ^18^F-JK-PSMA-7 (Dietlein et al. 2020). But there is an important difference between the population studied and ours. Indeed, in the sub-group of patients with PSA values > 2 ng/ml, the average PSA level was 159 ng/ml compared to our subgroup with an average PSA value of 10.26 ng/ml, indicating that we selected patients with a less abundant metastatic load. Also, the proportion of patients with PSA > 0.3 in which we detected positive lesions (36/38 = 95%) was very similar to the proportion found in the previous study (61/63 = 97%) (Dietlein et al. 2021).

So, globally, DR found in our study, making use of reduced doses and acquisition durations and a digital photon detection system, was in the range reported with the standard methodology. We maintain a late time of acquisition at 140 post-injection, in accordance with data from a previous study showing that the values for SUVmax and SUVpeak in the PSMA-positive lesions increased for up to 60 min after injection and remained at this intensity up to 140 min post injection (Hohberg et al. 2019). Obviously, shorter acquisition durations and reduced doses of tracers have multiple advantages, allowing a larger number of patients to be imaged at the similar cost and with a lower radiation burden on each patient.

Importantly, the detection of lesion in patients with very low PSA levels seem not to be affected by these optimized conditions. Indeed, the lowest PSA value associated with a positive ^18^F-JK-PSMA-7 PET/CT was 0.12 ng/ml in our study while it was 0.3 ng/ml in the previous study performed with the same tracer (Dietlein et al. 2021). Also, the tumour/background ratio of the PSMA positive lesions remain extremely favourable in our conditions of acquisition on the digital PET/CT.

The detection rate in our patient population did not differ significantly between the groups of patients with and without androgen deprivation therapy and chemotherapy after initial loco-regional treatment. The influence of the androgen deprivation therapy on PSMA expression remains controversial. This aspect was evaluated in some preclinical and clinical studies (Simaeys et al. 2021; Vaz et al. 2020; Ceci et al. 2021; Wondergem et al. 2017; Afshar-Oromieh et al. 2018).

In the preclinical study recently published by our group, a change in PSMA expression in the course of a standard therapeutic management of PCa was found. Translated in the clinical practice, the result calls for caution in the interpretation of reduced PSMA-based tracer uptake after successive ADT and chemotherapy since it may be related to downregulation of PSMA expression in dedifferentiated and rapidly proliferating tumor cells (Simaeys et al. 2021). Opposite to this view, the 2021 EANM standardized reporting guidelines mentions that androgen receptor (AR) inhibition may increase PSMA expression in PCa. The contributors to the guidelines therefore draw our attention to the risk of falsely reporting disease progression shortly after initiation of AR-targeted therapies. Their recommendation follows some reports of transiently increased PSMA uptake during the first weeks of hormonal blockade. Obviously, contrary effects may combine and lead to variable global response, since up- or downregulation of PSMA may accompany the actual therapeutic effect on cell viability (Ceci et al. 2021). Unfortunately the small population we studied, treated with a variety of therapeutic regimens, does not allow to differentiate the effects of the various compounds in use, in particular agonists and antagonists of the gonadotrophin-releasing hormone.

If ADT is going to influence PSMA expression, the temporal relationship between PSMA expression and initiation of ADT is a factor to consider. Since it is not controlled in most studies evaluating PSMA PET/CT imaging, it may participate to the variability of performance observed among the different studies (Vaz et al. 2020).

## Conclusions

Our study confirms ^18^F-JK-PSMA-7 PET/CT as a robust imaging method in PCa patients with BCR, even at low PSA values. Previous treatments with ADT after initial local ablation does not seem to affect the performance of the method. Digital technology for photon detection allows reduction of tracer dose and acquisition time without affecting performance of ^18^F-JK-PSMA7 PET/CT in this setting.

## Data Availability

The original contributions presented in the study are included in the article/supplementary materiel, further inquiries can be directed to the corresponding author.

## Abbreviations

PCa: Prostate cancer
BCR: Biochemical recurrence
DR: Detection rate

## References

[CR1] Afshar-Oromieh A, Debus N, Uhrig M, Hope TA, Evans MJ, Holland-Letz T, Giesel FL, Kopka K, Hadaschik B, Kratochwil C, Haberkorn U (2018). Impact of long-term androgen deprivation therapy on PSMA ligand PET/CT in patients with castration-sensitive prostate cancer. Eur J Nucl Med Mol Imaging.

[CR2] Ceci F (2015). (68) Ga-PSMA PET/CT for restaging recurrent prostate cancer: which factors are associated with PET/CT detection rate?. Eur J Nucl Med Mol Imaging.

[CR3] Ceci F, Oprea-Lager DE, Emmett L (2021). E-PSMA: the EANM standardized reporting guidelines v1.0 for PSMA-PET. Eur J Nucl Med Mol Imaging.

[CR4] Dietlein F, Hohberg M, Kobe C, Zlatopolskiy BD, Krapf P, Endepols H, Täger P, Hammes J, Heidenreich A, Neumaier B, Drzezga A, Dietlein M (2020). An (18)F-labeled PSMA ligand for PET/CT of prostate cancer: first-in-humans observational study and clinical experience with (18)F-JK-PSMA-7 during the first year of application. J Nucl Med.

[CR5] Dietlein M, Kobe C, Kuhnert G, Stockter S, Fischer T, Schomäcker K, Schmidt M, Dietlein F, Zlatopolskiy BD, Krapf P, Richarz R, Neubauer S, Drzezga A, Neumaier B (2015). Comparison of [(18)F]DCFPyL and [(68)Ga]Ga-PSMA-HBED-CC for PSMA-PET imaging in patients with relapsed prostate cancer. Mol Imaging Biol.

[CR6] Dietlein F, Mueller P, Kobe C, Endepols H, Hohberg M, Zlatopolskiy BD, Krapf P, Heidenreich A, Neumaier B, Drzezga A, Dietlein M (2021). [^18^F]-JK-PSMA-7 PET/CT under androgen deprivation therapy in advanced prostate cancer. Mol Imaging Biol.

[CR7] Ghosh A, Heston WD (2004). Tumor target prostate specific membrane antigen (PSMA) and its regulation in prostate cancer. J Cell Biochem.

[CR8] Hohberg M, Kobe C, Krapf P, Täger P, Hammes J, Dietlein F, Zlatopolskiy BD, Endepols H, Wild M, Neubauer S, Heidenreich A, Neumaier B, Drzezga A, Dietlein M (2019). Biodistribution and radiation dosimetry of [18F]-JK-PSMA-7 as a novel prostate-specific membrane antigen-specific ligand for PET/CT imaging of prostate cancer. EJNMMI Res.

[CR9] Rowe SP, Campbell SP, Mana-Ay M, Szabo Z, Allaf ME, Pienta KJ, Pomper MG, Ross AE, Gorin MA (2020). Prospective evaluation of PSMA-targeted 18F-DCFPyL PET/CT in men with biochemical failure after radical prostatectomy for prostate cancer. J Nucl Med.

[CR10] Van Simaeys G, Doumont G, De Maeseneire C, Passon N, Lacroix S, Lentz C, Horion A, Warnier C, Torres D, Martens C, Vierasu I, Egrise D, Goldman S (2021). [^18^F]-JK-PSMA-7 and [^18^F]-FDG tumour PET uptake in treated xenograft human prostate cancer model in mice. Eur J Nucl Med Mol Imaging.

[CR11] Surti S (2007). Performance of philips Gemini TF PET/CT scanner with special consideration for its time-of-flight imaging capabilities. J Nuclear Med.

[CR12] Vaz S, Hadaschik B, Gabriel M, Herrmann K, Eiber M, Costa D (2020). Influence of androgen deprivation therapy on PSMA expression and PSMA-ligand PET imaging of prostate cancer patients. Eur J Nucl Med Mol Imaging.

[CR13] Wondergem M (2019). Early lesion detection with 18F-DCFPyL PET/CT in 248 patients with biochemically recurrent prostate cancer. European J Nuclear Med Mol Imaging.

[CR14] Wondergem M, van der Zant FM, Knol RJJ, Lazarenko SV, Pruim J, de Jong IJ (2017). ^18^F-DCFPyL PET/CT in the detection of prostate cancer at 60 and 120 minutes: detection rate, image quality, activity kinetics, and biodistribution. J Nucl Med.

[CR15] Zhang J, Maniawski P, Knopp MV (2018). Performance evaluation of the next generation solid-state digital photon counting PET/CT system. EJNMMI Res.

